# The evolution of stereotactic radiosurgery for vestibular schwannomas: recent evidence and outcomes

**DOI:** 10.1007/s11060-025-05252-1

**Published:** 2025-10-14

**Authors:** Othman Bin-Alamer, Mahmoud Osama, Jason P. Sheehan

**Affiliations:** 1 Department of Neurological Surgery, Loma Linda University Medical Center, Loma Linda, CA USA; 2 Department of Neurological Surgery, University of Virginia Health System, 1215 Lee St, Charlottesville, Virginia VA22908 USA

**Keywords:** Vestibular schwannomas, Stereotactic radiosurgery, Gamma knife, Subtotal resection, Neurofibromatosis type 2

## Abstract

**Purpose:**

Vestibular schwannomas (VS) are benign tumors of the vestibular nerve, increasingly detected due to improved and more frequent neuroimaging studies. A summary of the evolution of radiosurgery for VS management is undertaken.

**Methods:**

We broadly review the history and current state of stereotactic radiosurgery in the management of vestibular schwannomas.

**Results:**

Management includes observation, microsurgical resection, and stereotactic radiosurgery (SRS). First used for VS in the late 1960s, SRS achieves > 90% tumor control with safe complication profile. In small VS, early SRS reduces tumor progression and the need for delayed intervention with better cranial nerve outcomes compared to observation. For large VS, SRS provides high tumor control with exceedingly lower complications than surgery in select cases. In neurofibromatosis type 2 (NF2), SRS offers durable tumor control, though hearing preservation and clinical outcomes varies as it contingent on the complex clinical presentation and disease burden, a unique challenge of NF2 patients. Comparative studies and randomized trials reinforce SRS as an effective, minimally invasive treatment with superior long-term tumor control and lower morbidity. Hearing preservation depends on cochlear radiation dose, tumor volume, degree of hearing at time of intervention, age, and tumor morphology. In NF2 and large VS, careful patient selection is critical.

**Conclusion:**

Stereotactic radiosurgery is playing an increasingly important role in the management of patients with vestibular schwannomas. Future research will refine dose optimization, patient selection, and long-term functional outcomes.

## Introduction

**Fig. 1:**
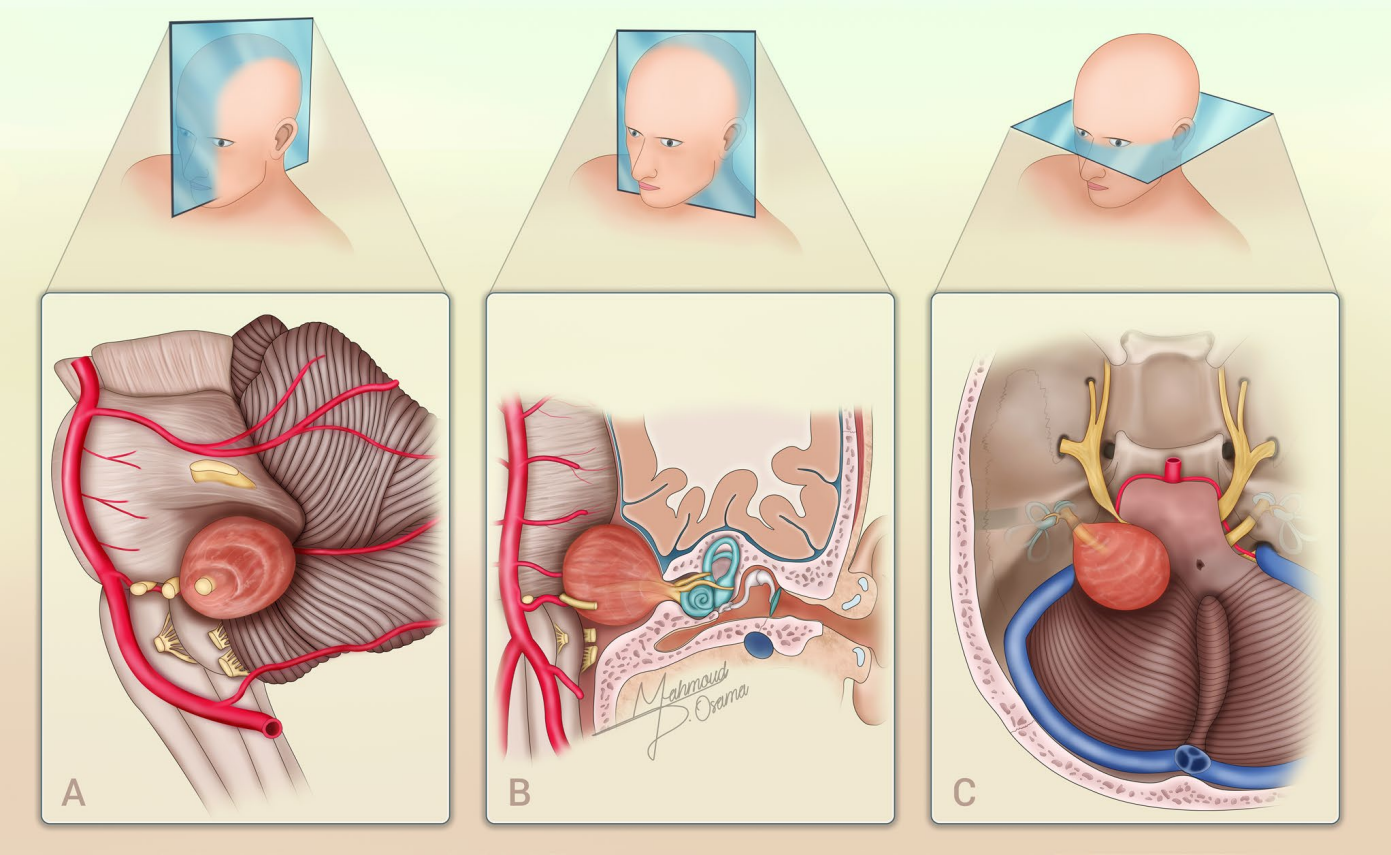
An illustration depicting a left-sided vestibular schwannoma across three views: sagittal (**A**), coronal (**B**), and axial (**C**) sections. Each section highlights the anatomical details and extent of the tumor

Vestibular schwannomas (VS) are benign, slow-growing neoplasms arising from the Schwann cells of the vestibular portion of the eighth cranial nerve, with an incidence of approximately 1.2 per 100,000 individuals per year (Fig. 1) [1–3]. 

Advances in neuroimaging have led to an increase in incidental diagnoses, with many VS detected at smaller sizes, often before the onset of significant neurological symptoms [4]. The clinical presentation varies based on tumor size and growth dynamics, with progressive sensorineural hearing loss being the most common initial symptom, followed by tinnitus, vestibular dysfunction, and, in larger lesions, brainstem compression or hydrocephalus [5]. 

Historically, VS management strategies mainly included observation and microsurgical resection, with observation being the preferred approach for small, asymptomatic VS. However, long-term volumetric studies have demonstrated that tumor progression was observed in up to 80% of cases within 5 years, with a 5-year progression-free survival rate of 20%, often necessitating delayed intervention [6]. While microsurgical resection offers definitive tumor removal, it carries a significant risk of cranial nerve morbidity, particularly facial nerve dysfunction and irreversible hearing loss. However, the advent of stereotactic radiosurgery (SRS) has become an established treatment modality for vestibular schwannomas by providing a minimally invasive alternative with high tumor control rates, reduced morbidity, and neurological preservation or, at times, improvement. The concept of SRS originated in 1951 with Lars Leksell, who made significant advancements in stereotaxy, to precisely target intracranial lesions with focused radiation, initially using orthovoltage X-rays and later transitioning to cobalt-60 gamma radiation with the development of the Gamma Knife [7]. The first reported application of SRS for VS occurred in the late 1960s by Leksell, with early VS SRS applications were primarily in patients with residual or recurrent disease following microsurgical resection [8, 9]. 

By the late 1970s and early 1980s, Leksell and his disciples—especially L. Dade Lunsford and others—leveraged advancements in imaging, particularly computed tomography (CT) and later magnetic resonance imaging (MRI), to improve tumor localization and treatment planning, enhancing the precision and safety of SRS [10–12]. Long-term studies from various centers in the US and across the globe demonstrated that single-session SRS could achieve tumor control rates often exceeding 90% while preserving cranial nerve function better than microsurgery in many cases [13–19]. These findings led to the widespread adoption of SRS, particularly in patients with small-to-medium tumors, elderly patients, or those with medical comorbidities. In addition, emerging approaches such as planned subtotal resection followed by adjuvant SRS and the increasing use of repeat SRS for progressive or recurrent tumors now complement primary treatment decisions. These developments reflect a growing emphasis on minimally invasive, function-preserving interventions and are increasingly shaping modern clinical practice. In this article, we explore the evolving role of SRS in the management of VS, summarizing recent evidence from contemporary clinical trials and landmark studies.

### Rationale and mechanism

The rationale for SRS is based on the slow-growing nature of VS, where a targeted radiation dose can induce tumor stabilization or regression without the risk of open surgery [20–24]. Given that observation often leads to eventual tumor progression and the need for delayed intervention, early SRS has been proposed as a proactive approach to prevent tumor growth and neurological deterioration while preserving hearing and facial nerve function [17, 25]. 

SRS exerts its effects through a combination of direct and indirect mechanisms that disrupt tumor cell proliferation and vascular integrity [20, 21]. High-dose radiation induces double-strand DNA breaks, leading to mitotic inactivation rather than immediate apoptosis [21–24]. Because VS cells are largely quiescent, cell death occurs gradually, with tumor growth arrest preceding any appreciable volumetric regression. In addition to direct cytotoxicity, radiosurgery disrupts the intratumoral microvasculature, triggering endothelial injury and progressive vessel occlusion [20]. This ischemic effect contributes to delayed tumor shrinkage and may explain why some VS initially demonstrate transient pseudoprogression on follow-up imaging before stabilizing or regressing over time. Reflecting these underlying mechanisms, Balossier et al. [26] evaluated the post-SRS dynamics of VS volume evolution and characterized five distinct volumetric patterns, including some showing pseudoprogression, advocating for no retreatment decision before 5 years post-SRS based on volume changes alone. In addition, pseudoprogression can be predicted using radiomics, which turns medical images into high-dimensional data, and machine learning. These models can be used to predict treatment outcomes for VS following SRS with accuracy ranging from 77% to 88.4%, helping distinguish tumor control from potential failure [27, 28]. 

**Fig. 2:**
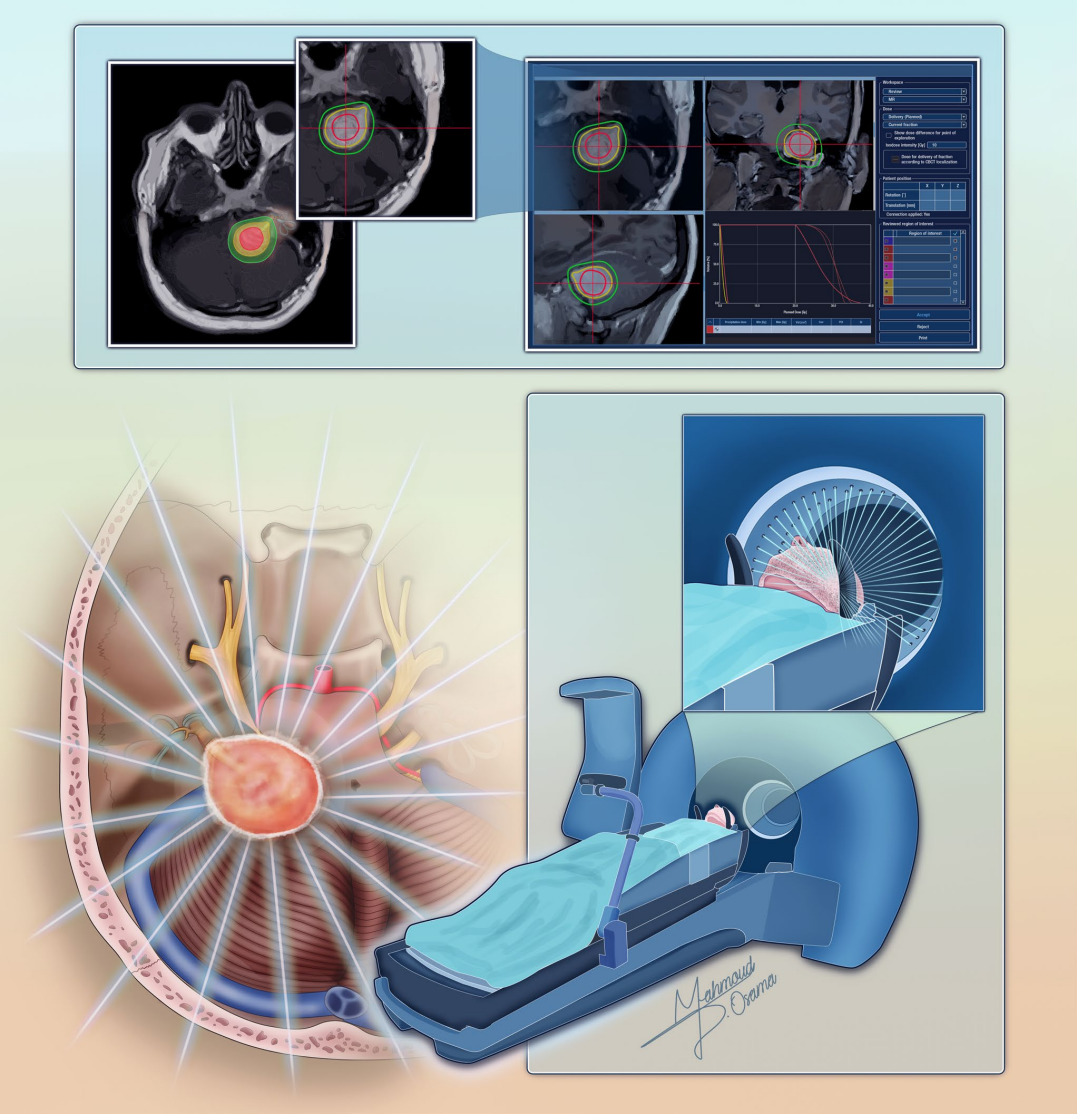
A schematic representation illustrating the process of gamma knife radiosurgery for vestibular schwannoma. The figure demonstrates the dose planning process and depicts the beams precisely targeting the tumor

Precise radiation delivery is critical to achieving tumor control while sparing adjacent cranial nerves. SRS modalities such as Gamma Knife (Fig. 2), CyberKnife, and LINAC-based systems use highly conformal dose distributions to minimize radiation exposure to surrounding structures.

Standard radiosurgical doses range from 11 to 13 Gy to the tumor margin, optimizing tumor control while reducing the risk of facial and trigeminal neuropathy. Cochlear dose remains a critical determinant of auditory outcomes. Tuleasca et al. [29] found that a mean cochlear biologically effective dose (BED) of ≤ 8 Gy was associated with better hearing preservation rates in patients undergoing SRS for VS, and Baschnagel et al. [30] found that patients who received a mean cochlear dose of less than 3 Gy had a higher rate of hearing preservation compared to those who received higher doses. It is a commonly accepted practice to adopt a marginal dose of 11 to 13 Gy, while striving to maintain the average cochlear dose to < 4 Gy [13–19, 31]. Advances in imaging guidance and artificial intelligence-enhanced treatment planning continue to refine radiosurgical strategies, with ongoing research focused on dose-response relationships and predictive models to optimize outcomes. These innovations are already being explored in practice, as demonstrated by Bossi Zanetti et al. [32] in developing radiomics-based predictive models for CyberKnife^®^-treated VS, Shapey et al. [33] in creating an AI framework for automated segmentation and volumetry using MRI, and Lee et al. [34] in applying AI to longitudinal imaging analysis after radiosurgery.

### SRS for small and medium vestibular schwannomas

Attention has been drawn to elucidate the efficacy of SRS specifically for small and medium VS, especially as observation – which is associated with eventual progression and delayed intervention – has been the typical clinical decision for these tumors [35]. Given their indolent growth, management strategies must balance the risks of intervention against the natural history of untreated tumors. Several studies provided critical insight into the limitations of wait and watch approach, reflecting a substantial change in management practices. **(**Table 1**)**. Régis et al. [31] conducted one of the earliest comparative analyses comparing wait-and-see approach versus SRS in intracanalicular VS over a mean follow-up of 43.8 ± 40 months. Among 47 patients under observation, 74% demonstrated tumor progression necessitating delayed intervention, while only 3% of the 34 patients who received upfront SRS required further intervention. Additionally, hearing preservation at 5 years favored the radiosurgery group (64% vs. 41%). Building on this, the V-REX randomized controlled trial provided the first high-level prospective evidence comparing upfront SRS to observation in small and medium VS (tumor volume < 2 cm in the cerebellopontine angle) [36]. The trial enrolled 100 patients and randomized them to either early SRS (*n* = 50) or observation (*n* = 50), with a primary outcome assessing volumetric tumor response over four years. At trial completion, tumor volume was significantly lower in the early SRS group compared to observation (V4:V0 ratio of 0.87 vs. 1.51, *P* = 0.002), indicating that early SRS effectively halted or regressed tumor growth. The need for secondary intervention was substantially reduced: only 6% of patients in the early SRS group required additional treatment (2% repeat SRS, 4% salvage microsurgery), whereas 42% of patients in the observation group required SRS due to tumor growth. Expanding on these findings, the VISAS multicenter study provided a further comparative analysis with long-term follow-up [17]. With a matched cohort of 125 patients per group and a median follow-up of 36 months, the study demonstrated superior 5- and 10-year tumor control rates for SRS (99% CI, 97.1%-100%, and 91.9% CI, 79.4%-100%) compared to observation (45.8% CI, 36.8%-57.2%, and 22% CI, 13.2%-36.7%; *P* < 0.001). In a focused analysis on the role of SRS in Koos grade I VS compared to observation, the VISAS-K1 study provided critical insights into treatment outcomes for this earliest stage of tumor presentation [25]. Among 142 matched patients with a median follow-up of 36 months, SRS demonstrated superior tumor control, with a 100% control rate at both 5- and 8-year follow-ups, compared to 48.6% and 29.5% in the observation group, respectively (*P* < 0.001). Together, these studies reinforce that, despite the slow growth of VS, the probability of progression remains high, and early intervention with SRS offers a more definitive control strategy. While early SRS reduces the risk of long-term tumor progression, it is important to consider that some intracanalicular tumors may demonstrate limited growth and remain asymptomatic without ever requiring treatment [37]. Furthermore, transient post-SRS volume increases due to pseudoprogression may not necessitate intervention, but could be misinterpreted as failure, potentially leading to unnecessary follow-up procedures and increased healthcare utilization [38]. 

Preservation of hearing function remains a critical factor influencing treatment decisions in these tumors. Régis et al. [31] reported that while tumor growth correlated with hearing deterioration in the observational group, proactive SRS improved the likelihood of maintaining functional hearing. This was later supported by the VISAS study, which showed no significant difference in hearing preservation between SRS and observation at 5 and 9 years (SRS 60.4% CI, 49.9%-73%, vs. observation 51.4% CI, 41.3%-63.9%, and SRS 27% CI, 14.5%-50.5%, vs. observation 30% CI, 17.2%-52.2%; *P* = 0.53) [17]. The V-REX study revealed that while both groups experienced hearing decline, there was no significant difference in pure-tone average deterioration at 4 years (18 dB in SRS vs. 20 dB in observation, *P* = 0.30; mean difference, − 2 dB; 95% CI, − 8 dB to 5 dB) [36]. At 4 years, the mean word recognition score was 42% points in the upfront radiosurgery group and 47% points in the wait-and-scan group. In Koos grade I VS, the VISAS-K1 study found that hearing preservation was comparable between groups, with serviceable hearing maintained in 70.1% of the SRS group versus 53.4% of the observation group at 5 years (*P* = 0.33) [25]. A more detailed evaluation of hearing preservation was provided by Akpinar et al., [39] who assessed patients with normal hearing at diagnosis. Their analysis demonstrated that early intervention (SRS within ≤ 2 years of diagnosis) resulted in significantly higher rates of long-term hearing preservation. At a median follow-up of 75 months, serviceable hearing was maintained in 88% of early-treated patients versus 55% in those undergoing delayed SRS (*P* = 0.006), while normal hearing was preserved in 77% versus 33%, respectively (*P* < 0.0001). These results indicate that delaying treatment may allow subclinical cochlear dysfunction to progress, leading to irreversible hearing loss even before radiologic tumor enlargement is apparent. Therefore, in patients with small-volume tumors (Koos I–II) and good hearing function, early SRS should be considered a favorable management strategy, balancing tumor control with functional preservation. On the other hand, the meta-analysis by Brito et al. [40] suggested that observation may offer superior hearing preservation over time in some cases, underscoring the importance of individualized management based on baseline auditory function and tumor characteristics. Cochlear radiation dose remains an essential determinant of auditory outcomes. Tuleasca et al. [29] demonstrated, in a univariable analysis, that a mean cochlear BED of ≤ 8 Gy was associated with better hearing preservation, while Schnurman et al. [41] in a propensity score matched analysis reported that higher cochlear dose exposure correlated with increased risk of hearing loss. Other factors that were found to be associated with better hearing outcomes are patient age, tumor morphology, hearing function at time of intervention, and tumor volume at presentation [17, 42]. 

SRS has been associated with a lower incidence of neurological morbidity compared to observation. In propensity score matched cohorts, the VISAS study found that SRS significantly reduced the odds of developing tinnitus (OR = 0.39, *P* = 0.01), vestibular dysfunction (OR = 0.11, *P* = 0.004), and any cranial nerve palsy (OR = 0.36, *P* = 0.003) [17]. Specifically in Koos grade I, SRS was associated with a reduced risk of tinnitus (OR = 0.46, *P* = 0.04), vestibular dysfunction (OR = 0.17, *P* = 0.002), and overall cranial nerve dysfunction (OR = 0.49, *P* = 0.03) at last follow-up [25]. However, it is important to recognize that radiosurgical failure, though infrequent, may necessitate salvage microsurgery, which may carry increased risk of hearing deterioration and facial nerve dysfunction particularly given the tumor progression [43]. Studies such as that conducted by Marinelli et al. [44] and Lee et al. [45] emphasize some this potential consequence due to difficulty of tumor resection from the facial nerve, highlighting the importance of careful patient selection when considering upfront SRS and others have recommended subtotal resection for patients with tumor progression after SRS. This underscores the need for a nuanced, patient-centered approach that weighs both the benefits of early tumor control and the risks associated with potential salvage interventions.

**Table 1:** Summary of key studies on SRS vs. Observation for small and medium vestibular schwannomas

Study	Design / Population	Koos Grade	Follow-up (months)	Tumor Control	Hearing Preservation	Secondary Intervention (%)	Key Findings
Régis et al. 2010 [31]	Prospective cohort; 34 SRS vs. 47 obs	Koos I (intracanalicular)	Mean: 43.8	97% (SRS) vs. 26% (obs)	5-yr: 64% (SRS) vs. 41% (obs)	3% (SRS) vs. 74% (obs)	Early SRS showed superior tumor control and hearing preservation.
Akpinar et al. 2016 [39]	Retrospective cohort; 57 early SRS (≤ 2 yrs) vs. 31 late SRS (> 2 yrs)	N/A	Median: 75	95% (early) vs. 90% (late); *P* = 0.73	Serviceable: 88% (early) vs. 55% (late), *P* = 0.006. Normal: 77% vs. 33%, *P* < 0.0001	N/A	Delayed SRS was associated with a higher risk of irreversible hearing loss.
Dhayalan et al., 2023 (The V-REX) [36]	RCT; 50 SRS vs. 50 obs	Koos I-II	48	V4:V0 ratio: 0.87 (SRS) vs. 1.51 (0bs); *P* = 0.002	PTA loss: 18 dB (SRS) vs. 20 dB (obs); *P* = 0.30	6% (SRS) vs. 44% (obs)	SRS effectively halted tumor growth at 4 years.
Bin-Alamer er al, 2024 (The VISAS Study) [17]	Multicenter retrospective matched cohort; 125 SRS vs. 125 observation	Koos I-II	Median: 36	5-yr: 99% (SRS) vs. 45.8% (obs) 10-yr: 91.9% vs. 22%; *P* < 0.001	5-yr: 60.4% (SRS) vs. 51.4% (obs) 9-yr: 27% vs. 30%; *P* = 0.53	N/A	SRS achieved superior tumor control and reduced odds of tinnitus, vestibular dysfunction, and cranial nerve palsy.

Abbreviations: SRS = stereotactic radiosurgery; obs = observation; PTA = pure-tone average; RCT = randomized controlled trial; V4:V0 = tumor volume ratio at year 4 to baseline; yr = year

### SRS for large vestibular schwannomas

Microsurgical resection remains the primary treatment for large VS causing mass effect, brainstem compression, or hydrocephalus. However, increasing evidence supports SRS as a viable alternative in selected patients **(**Table 2**)** [18, 46]. Pikis et al. [18] reported a multicenter retrospective analysis of 627 patients treated with primary, single-session SRS which similarly demonstrated high tumor control rates (94.1% at 3 years; 5- and 10-year actuarial control of 92.3% and 87.6%, respectively). Multivariable analysis showed that early post-SRS tumor expansion occurred in 10.7% of cases and was significantly associated with treatment failure (HR, 102.4; 95% CI, 34.7-302.2; *P* = 0.001). Post-SRS facial nerve function deteriorated in 3.0% of patients, and hydrocephalus requiring shunting occurred in 6.0%. A retrospective comparative study of 901 patients undergoing either microsurgical resection or SRS with a mean follow-up of 7 years demonstrated an incidence of recurrence of 7% after surgery, and 11% in the SRS group, with a significant Log-rank test (*P* = 0.031) [47]. However, functional outcomes and complications favored SRS, with lower rates of facial nerve palsy and similar hearing deterioration between groups. A meta-analysis of 10 studies including 1398 large VS patients underwent SRS reported an overall tumor control rate of 90.7% (95% CI, 86.3–94.4), with 2-, 6-, and 10-year Kaplan-Meier estimates of 96.0%, 88.8%, and 84.5%, respectively [46]. Long-term hearing preservation remained limited (56.5%), and hydrocephalus occurred in 5.6% of patients. Facial nerve preservation was 100% (95% CI, 99.9–100.0), underscoring the relative safety of SRS for cranial nerve function.

Margin dose is a key determinant of post-SRS complications. High-dose SRS (≥ 13 Gy) increases the risk of facial nerve dysfunction in large VS. In the Pikis et al. cohort, patients receiving ≥ 13 Gy had significantly worse facial nerve preservation rates compared to those treated with lower doses (Multivariable analysis, HR, 4.8; 95% CI, 1.2–19.3; *P* = 0.03) [18]. Post-SRS tumor expansion was another predictor of facial nerve deterioration (Multivariable analysis, HR, 5.0; 95% CI, 1.1–23.7; *P* = 0.04). Hypofractionated SRS (3–5 fractions) for large VS has also been explored as an alternative strategy with limited data suggesting possible reduced cranial nerve toxicity while maintaining tumor control [48]. For patients aged ≥ 65 years or those with comorbidities contraindicating surgery, SRS may be the preferred option. A multicenter retrospective study of 150 elderly patients treated with single-session SRS for Koos IV VS reported tumor control rates of 96.0% and 86.2% at 5 and 10 years, respectively [49]. Despite a lower 5-year hearing preservation rate (69.3%), facial nerve preservation remained high (98.7% at 5 years, 91.0% at 10 years). Hydrocephalus occurred in 8.0% of cases. These findings suggest that SRS is a safe and effective alternative especially in elderly patients, particularly when functional preservation is prioritized.

**Table 2:** Summary of key studies on SRS for large vestibular schwannomas

Study	Design / Population	Koos Grade	Follow up (months)	Tumor Control	Hearing Preservation	Secondary Intervention (%)	Key Findings
Pikis et al. 2022 [18]	Multicenter retrospective cohort; 627 pts; single-session SRS	Koos IV	Median: 38	3-yr: 94.1%; 5-, 10-yr: 92.3%, 87.6%, respectively	5-, 10-yr: 65%, 44.6%, respectively	6%	SRS provided safe, effective tumor control in selected Koos IV VS patients, with modest hearing preservation, low facial nerve morbidity, and hydrocephalus in 5.3% of cases.
Tatagiba et al. 2023 [47]	Bicentric retrospective cohort; 559 SRS vs. 342 surgery	Koos I, II, III, and IV	Mean: 78.38	89% (SRS) vs. 93% (surgery); *P* = 0.031	54% (SRS) vs. 37% (surgery); *P* < 0.001	62% (SRS) vs. 16% (surgery)	SRS achieved comparable control to surgery in small VS, but surgery offered superior control in large VS with differing morbidity and symptom improvement profiles.
Dumot et al. 2023 [49]	Multicenter retrospective cohort; 150 pts; single-session SRS	Koos IV	12	5-, 10-yr: 96.0%, 86.2%, respectively	5-, 10-yr: 69.3% and 50.9%, respectively	N/A	SRS offered high long-term tumor control and facial nerve preservation in elderly Koos IV VS patients, with 8% developing hydrocephalus and limited hearing preservation.
Szymoniuk et al. 2024 [46]	Meta-analysis study; 10 studies; 1398 pts	Koos IV	N/A	Overall: 90.7%; 2-, 6-, 10-yr: 96.0%, 88.8%, 84.5%, respectively	Overall: 56.5%; 2-, 6-, 10-yr: 77.1%, 53.5%, 38.1%, respectively	N/A	SRS provided high tumor control and facial nerve preservation with low tinnitus/vertigo risk, but carried hydrocephalus risk (5.6%) and suboptimal long-term hearing preservation.

Abbreviations: SRS = stereotactic radiosurgery; pts = patients; yr = year

### Stereotactic radiosurgery for neurofibromatosis type 2-associated vestibular schwannomas

Neurofibromatosis type 2 (NF2)-related VS presents a distinct therapeutic challenge due to complex presentation, bilateral nature, and progressive impact on hearing. SRS has emerged as a critical approach, aiming to balance tumor control with functional preservation [37]. Recent studies have delineated the efficacy and safety profile of SRS in NF2-associated VS [37, 50–52]. A large international multicenter study evaluated 328 tumors treated with SRS reported 10- and 15-year tumor control rates of 77% and 52%, respectively, with 85% freedom from additional treatment at 10 years [19]. Similarly, Shrivastava et al. [53] reported a tumor control rate of 72% with a 10-year survival rate of 71%, while Tosi et al. [52] conducted a systematic review highlighting an 88% tumor control rate across 16 studies, though with a noted decline in hearing preservation. Furthermore, Puataweepong et al. [54] demonstrated an 80% local tumor control rate at 10 years with a low incidence of non-auditory complications.

Hearing preservation remains a main concern in NF2 patients. Additionally, Kim et al. [55] observed an initial decline in hearing due to pseudoprogression in 29% of cases, but long-term stability was achieved in most patients. Tosi et al. [52] conducted a systematic review and meta-analysis demonstrating an 88% tumor control rate but noted significant hearing deterioration post-SRS, with an odds ratio of 0.26 for preserving serviceable hearing. These studies emphasize the need for individualized treatment approaches, balancing tumor control with functional outcomes.

The risk of radiation-induced malignancy remains a critical consideration in the long-term safety of SRS. Bin-Alamer et al. [19] reported no cases of radiation-associated malignancies in a large multicenter cohort. A systematic review by King et al. [55] found that malignant transformation of VS in NF2 patients post-radiosurgery is exceedingly rare, though isolated case reports have been reported [52, 56]. Given the available data, SRS remains a safe option, with malignancy risks being lower than previously assumed.

### Planned subtotal resection followed by SRS

Microsurgical resection remains a key approach for large VS with mass effect, brainstem compression, or hydrocephalus. However, the risks of complete resection, including facial nerve dysfunction and hearing loss, have led to the emergence of planned subtotal resection (STR) followed by SRS as an alternative strategy. This combination approach reduces the tumor volume, alleviates brainstem compression, and preserves cranial nerve integrity and function while leveraging the high tumor control rates and lower morbidity of SRS. Studies have shown that planned STR followed by SRS achieves long-term tumor control rates comparable to primary microsurgery while significantly reducing the risk of facial nerve dysfunction [57–60]. 

A study by Radwan et al., [59] analyzing 22 patients with Koos grade III-IV VS treated with STR and adjuvant SRS reported a 100% tumor control rate at 28 months follow-up, with 86% of patients maintaining excellent facial nerve function (House-Brackmann grade I-II). Similarly, Daniel et al. [60] evaluated outcomes in 32 patients undergoing planned STR followed by Gamma Knife radiosurgery, noting that 100% retained normal facial nerve function, and 77% maintained serviceable hearing after SRS. This approach typically allows functional outcomes comparable to those observed with primary SRS in small and medium-sized tumors while ensuring adequate tumor decompression in large VS. Similarly, Pan et al. [57] compared outcomes of intracapsular decompression followed by SRS versus radical extracapsular resection followed by SRS in a cohort of 35 patients. They found that 89% of patients in the decompression-plus-SRS group retained excellent facial nerve function, compared to only 35% in the radical resection group (*P* < 0.01). Additionally, all patients in the decompression group retained serviceable hearing postoperatively, whereas none in the radical resection group did (*P* < 0.001). These findings further validate the role of planned STR followed by SRS in optimizing functional outcomes in patients with large VS. Long-term tumor control following STR and adjuvant SRS is comparable to that of primary SRS for small tumors and superior to microsurgery alone for large tumors. Iwai et al. [61] reported an 86% five-year tumor control rate in a cohort of 40 patients undergoing STR with SRS, with facial nerve preservation in 95% of cases. Additionally, van de Langenberg et al. [58] reported a 90% radiologic tumor control rate in 50 patients treated with STR and subsequent SRS, with 94% retaining good facial nerve function. The emerging body of evidence supports planned STR followed by SRS as a viable alternative to radical resection alone, particularly in patients with large VS where complete resection poses significant risks.

### Repeat stereotactic radiosurgery for progressive vestibular schwannomas

While SRS achieves tumor control of >90% in VS, challenges managing the remaining < 10% progressive VS remain. Repeat SRS has emerged as an alternative to microsurgery, with growing evidence supporting its efficacy and safety for this challenging subset [62]. 

Kano et al. [62] reviewed six patients undergoing repeat SRS, reporting 100% tumor control at a median 29-month follow-up without new neurological deficits. Liscak et al. [63] observed tumor stability or regression in 22 of 24 patients, with 3 developing facial spasm, one having hearing decline, one with facial weakness, and one patient needing ventriculoperitoneal shunt. Yomo et al. [64] followed 15 patients for a median 64 months, showing 100% tumor control and six cases of significant regression without severe cranial nerve dysfunction.

Subsequent multicenter analyses reinforced these findings. Iorio-Morin et al. [65] studied 76 patients from the International Radiosurgery Research Foundation, reporting 98.6% two-year and 92.2% five- and ten-year tumor control. At the time of the first SRS, 30% of patients had useful hearing, which decreased to 8% after the second SRS and 5% at the last follow-up. Following the second SRS, 75% of patients experienced stable or improved symptoms. A deterioration in facial nerve function related to SRS was observed in 7% of cases. Khandalavala et al. [66] analyzed 32 patients across multiple institutions, demonstrating microsurgery-free survival of 97% at one year, 84% at three years, and 68% at five years.

A 2024 meta-analysis by Hajikarimloo et al. [67] (260 patients, 11 studies) reported a 91% pooled tumor control rate, 59% regression rate, and 36% serviceable hearing preservation. Cranial nerve toxicity remained relatively low (facial neuropathy 8%, trigeminal neuropathy 12%, adverse radiation effects 6%). Taori et al. [68] analyzed 18 patients with progressive VS post-primary SRS, reporting an 88% 10-year tumor control rate, with >80% symptom control (stable/improved) in various cranial nerve symptoms. The body of the current evidence demonstrates that repeat SRS provides high tumor control with low morbidity, delaying or avoiding microsurgical intervention in most cases.

### Limitations of the included studies

The studies included in this review were predominantly observational in nature, with many being retrospective cohort analyses. This inherent study design introduces potential for selection bias, which may lead to an overestimation of SR efficacy and limits the generalizability of reported outcomes. While prospective studies, such as the V-REX trial, offer more robust data and help mitigate some of these limitations, high-quality randomized controlled trials with long-term follow-up are still needed to provide definitive evidence regarding the role of SRS in this context. Furthemore, substantial clinical heterogeneity exists among patients with NF2, including variability in genetic mutations, age of onset, tumor multiplicity, and associated neurological deficits. Such diversity impacts both the natural history of the disease and the response to interventions like SRS. As a result, the applicability of outcomes drawn from more selectively chosen NF2 cohorts—often consisting of patients with preserved hearing or indolent tumor growth—may not extend to all phenotypic subgroups.

## Conclusion

SRS plays an increasingly important role in the management VS, offering high rates of tumor control with a generally favorable safety profile. For small tumors, early SRS reduces the likelihood of tumor progression and cranial nerve dysfunction, while offering comparable hearing preservation rates compared to observation. In selected patients with large VS, SRS—either alone or following planned subtotal resection—can achieve effective tumor control while reducing the risk of cranial nerve morbidity associated with microsurgery. In NF2, SRS provides durable tumor control in many cases, though hearing and cranial nerve outcomes are more variable and depend on baseline function and disease burden. Repeat SRS is a reasonable option for tumor progression following initial treatment and has been shown to help avoid or delay surgical intervention in the majority of treated patients. Clinical outcomes are influenced by cochlear dose, tumor volume and anatomic/morphological attributes, hearing/cranial nerve status at presentation and timing of SRS intervention. However, much of the current evidence stems from retrospective observational studies with inherent selection bias and limited generalizability, particularly in NF2 populations characterized by substantial clinical heterogeneity. Looking ahead, future directions include dose refinement to minimize cochlear and cranial nerve toxicity, AI-driven radiosurgical planning, and the use of radiogenomic and radiomic tools for personalized, data-driven VS management. Recent advances in these areas are already demonstrating promising potential to enhance treatment precision, optimize outcomes, and guide more nuanced patient selection in clinical practice.

## Data Availability

No datasets were generated or analysed during the current study.
